# Traffic Safety Perception, Attitude, and Feeder Mode Choice of Metro Commute: Evidence from Shenzhen

**DOI:** 10.3390/ijerph17249402

**Published:** 2020-12-15

**Authors:** Yuanyuan Guo, Linchuan Yang, Wenke Huang, Yi Guo

**Affiliations:** 1 Department of Geography and Resource Management, The Chinese University of Hong Kong, Hong Kong 999077, China; guoyuanyuan@link.cuhk.edu.hk; 2 Department of Urban and Rural Planning, School of Architecture and Design, Southwest Jiaotong University, Chengdu 611756, China; 3 The Research Center for Artificial Intelligence, Peng Cheng Laboratory, Shenzhen 518000, China; hwk727@163.com; 4 Department of Geography, Hong Kong Baptist University, Hong Kong 999077, China; 18481744@life.hkbu.edu.hk

**Keywords:** traffic safety, attitude, perception, objective factor, subjective factor, dockless bike-sharing, vehicle-related crash, last mile, multinomial logistic, Shenzhen, China

## Abstract

Like many other transit modes, the metro provides stop-to-stop services rather than door-to-door services, so its use undeniably involves first- and last-mile issues. Understanding the determinants of the first- and last-mile mode choice is essential. Existing literature, however, mostly overlooks the mode choice effects of traffic safety perception and attitudes toward the mode. To this end, based on a face-to-face questionnaire survey in Shenzhen, China, this study uses the two-sample t-test to confirm the systematic differences in traffic safety perception and attitudes between different subgroups and develops a series of multinomial logistic (MNL) models to identify the determinants of first- and last-mile mode choice for metro commuters. The results of this study show that: (1) Walking is the most frequently used travel mode, followed by dockless bike-sharing (DBS) and buses; (2) Variances in traffic safety perception and attitude exist across gender and location; (3) Vehicle-related crash risks discourage metro commuters from walking to/from the metro station but encourage them to use DBS and buses as feeder modes; (4) DBS–metro integration is encouraged by the attitude that DBS is quicker than buses and walking, and positive attitudes toward the bus and DBS availability are decisive for the bus–metro and DBS–metro integration, respectively; and (5) Substantial differences exist in the mode choice effects of traffic safety perception and attitudes for access and egress trips. This study provides a valuable reference for metro commuters’ first- and last-mile travel mode choice, contributing to developing a sustainable urban transport system.

## 1. Introduction

Cities are now encountering a large number of transport-related problems (mainly attributed to the extensive use of private cars or car dependency), including traffic congestion, deteriorated traffic safety situation, air pollution, increased vehicle emissions, environmental degradation, and excessive consumption of natural sources. “Reclaiming the city from cars” has constantly been advocated. Some of these vexing problems (e.g., air pollution) even adversely affect the population’s health [1,2,3]. Additionally, as a sustainable travel mode, transit (e.g., high-speed rail, metro, commuter rail, light rail, tram, bus rapid transit, and conventional bus transit) provides a high-capacity, medium-/long-distance, and low-emission transport service for residents. It contributes to overcoming car dependence and redressing a wide variety of contemporary cumbersome urban problems [4]. Hence, transit has received immense popularity and gained substantial interest in recent years and has also been promoted in a host of cities worldwide to facilitate people’s sustainable travel [5,6,7]. However, in general, it offers stop-to-stop services (rather than door-to-door services) and thus cannot cover every location of a city [8]. Poor transit accessibility, particularly in periphery areas, makes it hard, if not impossible, to reach a goal of offering convenient transport services, thereby generating first- and last-mile challenges for commuters [9,10]. 

The metro is a popular transit mode implemented in a multitude of cities, particularly large cities. Many motorized (e.g., private car, bus, and taxi) and non-motorized (e.g., walk, bicycle, and scooter) modes have been encouraged to serve as the feeder modes of the metro (or first-/last-mile modes before/after riding the metro), thereby attracting more commuters to use the metro. In a great many car-oriented U.S. metropolitan areas, park-and-ride is preferable, due in part to high car ownership and poor transit service [11]. In European cities where cycling is pervasive and even viewed as a cultural norm, bike-and-ride has gained enormous popularity [12]. By contrast, East Asian cities such as Shanghai and Hong Kong tend to encourage the bus (e.g., feeder bus and public bus) as a way of connecting the metro [13,14]. In addition to these traditional feeder modes, new types of shared mobility services, such as bike-sharing and ride-sourcing, have recently been introduced to promote metro use. For example, Ma et al. [15] suggest that for a 10% increase in bike-sharing ridership, Metrorail ridership increases by 2.8% in Washington. The majority of dockless bike-sharing (DBS) bikes are distributed around metro stations in Chinese cities [16]. These newly shared mobility services contribute to solving the first- and last-mile problem [17]. 

However, the decision-making process of the feeder mode choice is complicated and is determined by various factors. Previous studies offer insights into why individuals use specific transfer modes to connect transit [18]. They mainly focus on “hard” factors such as socio-economic and demographic characteristics, mandatory policies or requirements (e.g., wearing a helmet), and the physical environment such as topography, weather conditions, and the built environment at the origin and destination and around metro stations [7,19,20,21,22,23]. For instance, if parking space is available, people with cars and young adults with bicycles will be likely to drive or ride, respectively, to transit stations, [7,19]; transfer distance is fundamental to mode choice because different transfer modes usually correspond to different distance ranges (e.g., if the walking distance to a transit station goes beyond a certain threshold, residents will not choose to walk to transit) [23]; cycling-related facilities, such as sheltered parking spaces and bicycle lanes, contribute to a bike-friendly environment, thereby attracting bike-sharing–metro integration [24]. Besides, “soft” factors on the psychological aspect, such as attitude and perception, are postulated to determine travel behaviors, which has prominently been discussed in socio-psychological theories such as the theory of planned behavior (TPB) [25]. Traditional travel behavior literature also recurrently suggests that traffic safety and individual attitudes are crucial in shaping travel mode choice (e.g., [18,19]). The perceived traffic safety/risk affects the choice of self-controlled travel modes, such as driving and cycling [26,27,28]. The role of attitudes in mode choices could be as important as or even more important than the physical environment and socio-economic characteristics [29,30,31], but this is still inconclusive. For example, individuals with favorable attitudes toward a specific travel mode may likely use that mode [18,32]. Even though the effects of traffic safety perception and attitudes on mode choice are widely acknowledged, to the knowledge of the authors, limited studies have investigated (1) the impacts of the two psychological factors on the feeder mode choice of the metro; and (2) how the impacts vary across metro commuters’ first- and last-mile trips. 

To this end, based on a case study of Shenzhen, China, this study explores how the perceived traffic safety and the attitude toward transfer modes correlate to the feeder mode choice of metro commuters. A field questionnaire survey was conducted at many metro stations in Shenzhen, in which the metro commuter’s transfer mode for the access/egress trip has been examined. The two-sample t-test is used to confirm the systematic differences in traffic safety perception and attitudes between different subgroups, and a series of multinomial logistic (MNL) models is developed to identify the determinants of first- and last-mile mode choice for metro commuters. The study aims to address the following four research questions: (1) What is the mode share of access/egress trips in today’s Chinese mega cities? (2) Do traffic safety perceptions and attitudes vary by gender and location? (3) Are perceived traffic safety and attitudes toward transfer modes important in determining the transfer mode choice? (4) Are the effects of perceived traffic safety and attitudes toward the mode different between access and egress trips? We believe that this study contributes to the promotion of green and healthy urban mobility and serves as a valuable reference for Chinese mega cities and other settings with similar traffic conditions. 

The remainder of the paper is organized as follows. Section 2 introduces existing feed modes. Section 3 reviews the literature on the effects of perceived traffic safety and attitudes on feeder mode choice. Section 4 presents the study context, questionnaire survey, and methodologies. Section 5 offers the analyses of feeder mode share, the individual variance in perceived traffic safety and attitude, and the modeling results. Section 6 provides policy implications, research limitations, and avenues for future research. The final section (Section 7) concludes the paper.

## 2. Summary of Existing Feed Modes

Walking, a travel mode with economic, environmental, social, and health impacts [10,33], is often used to reach a transit station (walk-and-ride). However, it requires the use of the human body as a travel machine. Therefore, it is significantly restricted by travel distance [21]. Generally, the willingness to walk can be well described by distance decay curves: after 800–1000 m, the mode share of walking declines sharply. 

The bicycle’s integration with transit (bike-and-ride) is also common for commuters. The synergy of the bicycle and transit for access/egress trips can be summarized into three patterns: bicycle-and-transit, transit-and-bicycle, and bicycle-on-transit (taking a bicycle on transit) [34,35]. There are many preconditions for the bicycle-and-transit and transit-and-bicycle patterns, such as the ownership of bicycles, available secure parking spaces, and parking facilities (e.g., shelter and parking dock) around transit stations [36]. However, the bicycle-on-transit pattern is usually constrained by the parking space or capacity on transit, which may bring conflicts between regular transit passengers and bicycle–transit users [35]. Recently, bike-sharing services, including docked and dockless programs, have been introduced to various cities (e.g., Paris, Singapore, and Shanghai). This makes the bike–metro integration smarter, greener, and more economical because there is no need to bring bikes on transit and worry about the issues of theft and maintenance [16,37]. 

As for motorized feeder modes, the motorcycle (two-/three-wheeled motor vehicle), which usually carries 1–2 passengers, has a similar function with the taxi in many developing countries, such as China, Thailand, Vietnam, and India [38,39]. Motorcycle drivers always wait at large-scale residential areas and the exits of transit stations and solicit passengers. They often ride through narrow spaces during traffic jams. Thus, as for passengers, the motorcycle is fast (or time-saving) and easy to access. Moreover, in larger metropolitan areas, feeder buses, which are commonly operated in two forms—demand-responsive transit and fixed-route transit [40]—have been introduced by transit agencies to residents, especially those with poor transit accessibility. They perform well when the to-transit distance is long [22]. 

The private car is an option to access transit stations, and it involves two manners: park & ride and kiss & ride (i.e., passenger drop-off) [21]. The availability of park & ride facilities is a key factor for commuters with cars [20]. However, using the private car as the feeder mode of transit, particularly for the park & ride pattern, is more common in remote locations than downtown [41].

Other modes such as traditional and electric scooters, e-bike, taxi, and ride-hailing can serve as the feeder modes of transit. However, few empirical studies have discussed their integration with transit [42].

## 3. Related Studies

### 3.1. Perceived Traffic Safety and Last-Mile Mode Choice

Traffic safety is usually measured by how unlikely accidents occur. It is reported that traffic accidents kill about 1.2 million people all over the world each year [43]. Traffic safety is determined by many factors, such as road characteristics, climate and weather, and—perhaps most importantly—vehicle speeds [44]. Previous research confirmed that the higher the speed, the greater the possibility of traffic accidents [28,45]. 

Perceived traffic safety refers to individuals’ perceived likelihood of an accident-free traffic outcome (i.e., avoiding traffic accident and crash) [43,46]. It varies from person to person based on their background (information and experience) and how they deal with risks [47,48]. For example, Salonen indicated that men have a higher level of traffic safety awareness, in-vehicle security, and emergency management than women [43]. Bordagaray et al. confirmed that young adults (aged 34 years or below) perceive traffic safety as less important than older people do [1]. Moreover, the built environment is associated with people’s safety perceptions. Intersection density and the presence of major road crossings en-route were found to insensibly affect the individual’s perception of safety, such as the fear of collision [28]. Safety concerns also come from heavy traffic, such as high volume/speed of vehicles on streets [49,50]. 

Furthermore, the perception of traffic safety may significantly affect mode choice decision [51]. According to the TPB model, perceived behavior control, which means the perceived difficulty in or ease of performing a behavior, is one of the socio-cognitive factors determining the individual’s behavioral intention [25]. Moreover, a model for passenger transport developed by Van Wee [52] summarizes elements shaping travel behavior, including activity locations, transport resistances, needs, opportunities, and abilities. Typically, travel resistance consists of time, money, and other non-monetary costs, such as the perceived risk of traffic. Specifically, the perception that a certain type of transport mode is unsafe can be a psychological barrier to its use [53]. According to some empirical studies, the number of occurred crashes can directly affect the safety perception of pedestrians and bicyclists [54], thereby influencing active transport behaviors. Aziz et al. [55] also indicated that decreasing traffic crashes on pedestrians and bicyclists led to an increase in the likelihood of walking and cycling behavior. A recent study focusing on new safety challenges that autonomous vehicles (AVs) introduce indicates that, among road users, cyclists have the lowest level of perceived safety, followed by pedestrians and drivers, when their activities are near an AV [56]. 

### 3.2. Attitude and Last-Mile Mode Choice

Attitude can be defined as ‘‘global and relatively stable evaluations that people do about persons, things or ideas” [57]. Thus, attitudes involve positive or negative views that people have in terms of any aspect of reality [32,58]. According to existing literature [50,52,53], travel-related attitudes are usually connected to preferences for destinations, routes, activities, and modes of transport. A more general understanding of travel-related attitudes may also correlate with the individual’s beliefs (e.g., environmentalism) [59]. 

Some behavior theories, such as the theory of reasoned action (TRA) and its extension, namely the TPB, emphasized that the individual’s travel behavior is significantly influenced by attitudes. In TRA and TPB models, attitude is a predictor of the individual’s behavioral intention, which is a predictor of behaviors [25,60,61]. Travel behavior literature has well recognized the role of attitude in shaping travel behavior [32,62,63]. In particular, the individual’s attitude toward travel modes is evidenced to affect mode choice. For example, positive attitudes toward walking and cycling are related to frequent physical activities (through active travel), discouraging the motorized mode usage (e.g., car and bus) [32]. Thøgersen [64] found that a good attitude toward transit can predict transit use among Danish residents based on a panel survey during 1998–2000. Tran et al. [59] investigated the specific attitudes toward cars and buses and how such attitudes affect mode choice. They concluded that attitudes toward car use significantly affect the bus utility. Through a qualitative study in Porto (Portugal), Beirão and Sarsfield Cabral [65] also demonstrated the significant role of attitude in influencing the mode switch (from the car and public transit) for commuters. They suggested that improving the levels or images of public transit services could be effective to attract occasional public transport users and car users. Additionally, the relative attitudes among transport modes play a crucial role in affecting mode choice. He and Thøgersen [66] revealed that people’s favorable attitudes toward transit (relative to cars) make transit more attractive. In a similar vein, people who prefer cars to public transport tend to travel by car. 

In addition to the specific attitude toward the travel mode, the travel behavior effects of general attitudes with a broad concept have been studied. For example, a positive attitude toward physical activity promotes bicycling and walking behaviors [67]. Based on samples of Swedish commuters, Johansson et al. [27] found that attitudes toward flexibility and comfort influence mode choice. Additionally, some studies incorporated attitudes to analyze the influences of environmental awareness and sustainability concerns about mode choice and demonstrated the role of these attitudes [32,68]. 

However, studies on the effect of traffic safety and attitude toward mode choice are still scarce in terms of the specific condition of feeder trips. Previous studies have mostly investigated mode choice for general trips but paid limited attention to the first- and last-mile trips. Therefore, more sophisticated analyses are indispensable to explore the associations between traffic safety, attitude, and feeder mode choice.

## 4. Data and Methodology

### 4.1. Study Area: Shenzhen

**Figure 1:**
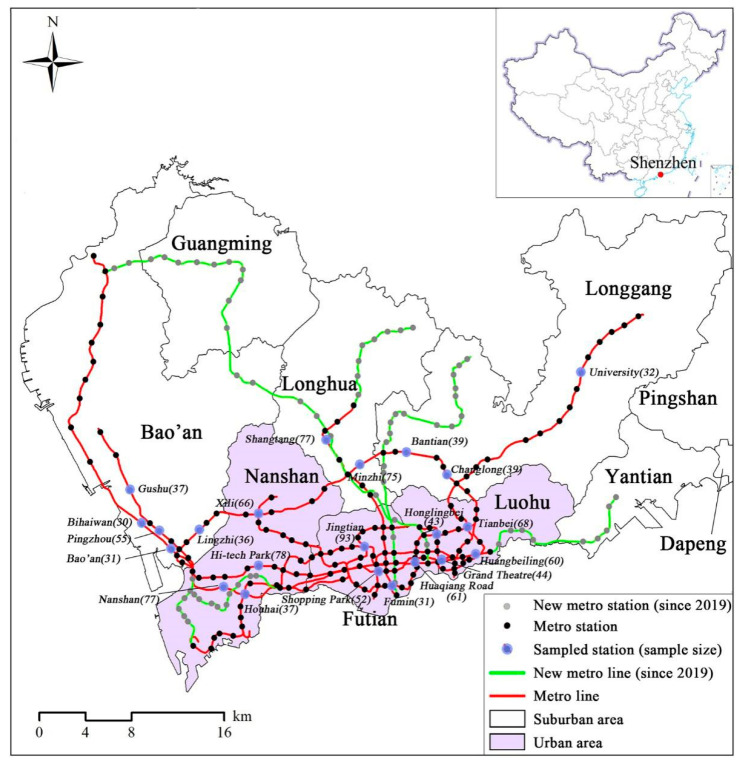
Study context of Shenzhen. Note: Since 2019, 70 new metro stations and 114.3 km metro lines have been built [37].

Shenzhen, a famous international metropolis located on the southern coast of China and adjacent to Hong Kong, is selected as our study area. In 2019, Shenzhen had a population of 13.44 million and covered an area of 1997 km^2^, indicating a high population density (6730 people/km^2^). Shenzhen had a vast amount of GDP in 2019 (390.34 billion dollars), ranking third in China. Over the last ten years, Shenzhen has widely been known as one of the Tier 1 cities in China (the other three cities are Beijing, Shanghai, and Guangzhou) [69]. Compared to other Asian modern cities, the GDP of Shenzhen is only smaller than Tokyo but larger than Singapore, Hong Kong, and Seoul. Additionally, Shenzhen has a large-scale metro system, which has opened since 28 December 2004. As of October 2020, Shenzhen has 11 metro lines, 237 metro stations, and a total mileage of 411 km (Figure 1). The daily ridership in 2018 was 5.14 million [37], ranking fourth in Mainland China. It is comparable to Hong Kong and Seoul but falls behind Tokyo. Therefore, Shenzhen is a representative modern city in China and also in East Asia.

The metro is an important travel mode for commuters, accounting for over 40% of trips taken by residents [37]. According to an online report issued by the Shenzhen Rail Transit Construction Headquarters Office, the average daily metro ridership and the per-km passenger volume in 2019 were 5.57 million and 19.2 thousand, respectively (Top Five in Mainland China) [70]. After the city has officially been designated as a “Transit Metropolis (*gongjiao dushi*)” by the Ministry of Transport of China, the local government has implemented a series of policy measures, such as establishing bus stops and allocating shared bikes (i.e., public bicycle and DBS) around metro stations, to promote the seamless connection between the metro and its feeder modes. 

Although Shenzhen witnessed an accomplished development of public transit, it experienced a sharp growth of private cars over the last decade. As of 2018, the number of motor vehicles in Shenzhen was approximately 3.37 million, and the total length of the road was 6443 km. This means that the density of motor vehicles (522.58 vehicles/km) is very high, which may result in an increased risk of traffic safety issues such as pedestrian/bicycle/vehicle-related crashes. Obviously, traffic safety issues are more acute in metro catchment areas with concentrated populations and vehicles than in other areas. 

### 4.2. Data and Variables

#### 4.2.1. Data

As formerly mentioned, this study aims to identify factors influencing the feeder mode of the metro, paying special attention to perceived traffic safety and attitude; and feeder mode includes walking, DBS, private bicycles, buses, taxi/Didi, cars, scooters, and other modes. We, therefore, collected the data by conducting a field questionnaire survey at 22 randomly selected metro stations in Shenzhen, China, between October and November in 2019 (Figure 1). During the survey, rainy days were excluded. Metro users who entered or left from the metro station during 7:30 a.m. and 9:00 a.m. were randomly selected as the survey samples. Given that metro commuters are usually in a hurry during the morning peak time, the respondents were requested to keep a leaflet with a quick response (QR) code. Thus, metro passengers can complete the questionnaire in their leisure time by scanning the QR code.

The questionnaire was composed of several parts, including transfer mode choice, the individual’s perceptions of and attitudes toward specific transport modes, and socio-demographic characteristics. In this study, traffic safety concerns were measured in a subjective way by considering two safety issues: pedestrian–bicycle crash and pedestrian/bicycle–vehicle crash. Metro users’ attitudes toward typical first-/last- mile travel modes were investigated. Additionally, relative attitudes of metro users, such as DBS versus walking and DBS versus bus, were recorded. Furthermore, questions on the attitude toward metro users’ ability to access DBS and buses and the attitude toward daily physical activities were asked. 

Questions related to traffic safety perception were designed by referring to the “Neighborhood Environment Walkability Scale (NEWS)” questionnaire, which includes questions on individuals’ perceptions of the environment [71]. Based on the NEWS, a four-point Likert scale (i.e., “strongly disagree” = 1, “somewhat disagree” = 2, “somewhat agree” = 3, and “strongly agree” = 4) was adopted to assess the perceived traffic safety. Similarly, individual’s attitudes were evaluated by the four-point Likert scale, thereby making the results comparable to those of perceived traffic safety. 

We received 1702 questionnaires, 1167 of which were valid (valid rate = 68.57%). The number of valid questionnaires for each metro station ranged from 30 to 93 (Figure 1). Among valid samples, 1086 respondents chose walking, DBS, or buses in access trips, while 1108 adopted one of these three modes to finish their egress trips. These three popular feeder modes account for 93.06% and 94.94% of the total trips in the access and egress scenarios, respectively, while other modes (e.g., private bicycle, taxi, Didi, and scooter) constitute less than 7%. 

#### 4.2.2. Variables

Table 1 shows the measurements and descriptive statistics of perceived traffic safety variables, attitude variables, and socio-demographic characteristics. 

The average score of bicycle crash is approximately 3 (“somewhat agree”), which is modestly larger than vehicle crash. This observation indicates that the bicycle-related crash could be a major safety concern for metro users in first-/last-mile trips and may significantly affect metro commuters’ first-/last-mile mode choice. Similarly, metro commuters may face a risk of crashes with vehicles along their feeder routes. As for the transport mode, metro riders usually have a positive attitude or fondness toward non-motorized modes (including walking and cycling) but a negative attitude toward the bus (the average score is only 2.3). 

As for the access trip connecting home and the metro station, DBS/bus availability involves how easy to find DBS bikes/bus stops around their home. In terms of the egress trip connecting the metro station and the workplace, DBS/bus availability means that how easy to find DBS bikes/bus stops around metro exits. However, we observe that many metro users agree that it is not easy to find a DBS bike for both access and egress trips. By contrast, metro users hold an attitude that the bus stop is relatively easy to access. Moreover, respondents usually want to have some daily physical activities. 

Table 1 indicates that more male passengers (59%) participated in the survey than female passengers (41%); most respondents were young (84% aged 35 years or below), well-educated (88% hold a bachelor degree or above), and middle-income earners (68% earn between 5000 and 14,999 RMB monthly); the respondents usually did not own a bicycle; and minimal differences in socio-demographic characteristics existed between the two scenarios. However, most access trips occurred in suburban areas (56.54%), while egress trips were largely concentrated in urban areas (79.69%). This observation indicates the jobs-housing imbalance in Shenzhen: Many metro commuters live in suburban areas because of low housing rent but work in urban areas with more job opportunities. 

The feeder trip distance is calculated via a geographic information system by connecting the geo-coded home/workplace addresses and the metro station reported by the respondents. The result shows that metro users have a longer transfer distance of home–metro feeder trips (access trip, 766 m) than that of the workplace–metro feeder trips (egress trip, 565 m).

### 4.3. Methodology 

This study applies two analysis approaches, namely the two-sample (two-tailed) t-test and the multinomial logistic (MNL) model. 

First, the two-tailed t-test is widely used to determine the statistical significance of difference between the means of two groups. In this study, it was performed to identify the variance in traffic safety perception and attitude among groups segmented by gender and location (male vs. female groups, and passenger groups working/living in urban areas vs. those working/living in suburban areas). 

Second, the MNL model is a popular method to relate a nominal (or categorical) outcome variable to its predictors [72]. One of its assumptions is that the random components of the utilities of different choices (error terms) are independent and identically distributed according to a Gumbel distribution (extreme value distribution). Moreover, the MNL model has the property of proportional substitution across alternatives (e.g., independent from irrelevant alternatives, or IIA) (recall the red bus-blue bus problem). In this study, feeder mode choices are typically nominal outcomes (with no natural ordering) and composed of three categories (i.e., walking, DBS, and bus). Thus, the MNL model fits well with our research and thus was used to identify how the factors of perceived traffic safety and attitudes are associated with first-/last-mile mode choice under two scenarios of access and egress feeder trips. More information on the MNL model can be found in [73]. 

Two kinds of transfer trips, namely access trips and egress trips, were considered. Thus, two MNL models (*Access* MNL model and *Egress* MNL model) are developed. As the name explicitly states, the *Access* MNL model scrutinizes the determinants of feeder mode choice for access trips, while the *Egress* MNL model does so for egress trips. Additionally, collinearity was assessed by calculating the variance inflation factor (VIF), and the result shows that the VIF values of all variables were less than 5.

## 5. Results

### 5.1. Feeder Mode Choice of the Metro

**Figure 2:**
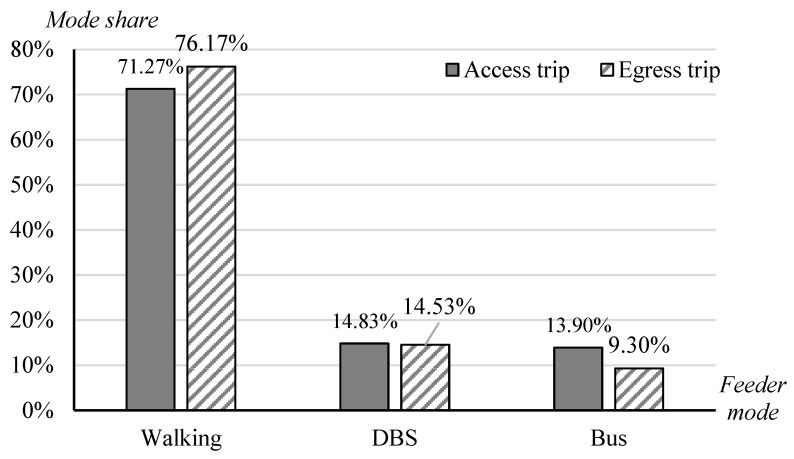
Share of the three feeder modes.

Figure 2 presents the share of the three feeder modes. It suggests that walking is the most frequently used mode by metro commuters for first- and last-mile trips, which is in line with many previous studies [22,23,34]. For either access or egress trips, the mode share of walking (more than 70%) is far larger than DBS (approximately 15%), closely followed by buses (about 10%). The relatively high share of DBS as the feeder mode of the metro demonstrates the popularity of DBS–metro integration in the study context. Compared with the share of the traditional docked bike-sharing (public bicycles) (4.4% in Nanjing [22] and 7.04% in Beijing) [23], DBS accounts for a larger market share, which reveals that it outperforms public bicycles in serving as the feeder mode of the metro. Furthermore, walking is more commonly adopted in egress trips than in access trips (difference in mode share = 5%). Few differences are observed between the two scenarios for DBS–metro integration, while metro users have a higher willingness to transfer by buses for home-metro connection than workplace-metro connection.

Table 2 reveals the feeder mode choice of men and women. It indicates that feeder mode choices substantially differ across genders. The male group has a similar share of walking with the female group but a higher share of DBS and a lower share of buses for access trips. As for egress trips, the differences in the share of three feeder modes between the two groups are subtle.

Table 3 shows the feeder mode choice in urban and suburban areas. Walking is preferable for metro users who live/work in urban areas than those living/working in suburban areas. A possible explanation for this observation is that the metro transit system is less developed in suburban areas, thereby generating a longer transfer distance unsuitable for walking but suitable for riding buses. 

### 5.2. Variance in Perceived Traffic Safety and Attitude 

Ample evidence shows that traffic safety perception is associated with gender [56,74] and location [75]. We, therefore, considered the variance in perceived traffic safety across genders and home/workplace locations. However, the variance in attitudes is usually correlated with socio-demographic characteristics, such as gender [43], so we made a comparison between male and female groups. We calculated the mean difference along two dimensions (gender (female versus male) and location (urban versus suburban areas)) and conducted two-sample t-tests. The mean differences were obtained as follows: male metro users minus female metro users, and metro users with a home/workplace located in urban areas minus those with a home/workplace situated in suburban areas.

Table 4 shows the result of the two-sample t-test. The perceived traffic safety significantly varies across gender and location. Compared with male metro users, female counterparts perceive a higher risk of vehicle-related crashes but a statistically equivalent risk of bicycle-related crashes. Moreover, the perceived risk of bicycle and vehicle crashes could be more obvious in suburban areas than in urban areas for access trips (connecting the home and the metro), but the reverse is true for egress trips (connecting the workplace and the metro). 

Table 4 reveals no significant difference in attitudes toward feeder modes (i.e., cycling, walking, bus, and DBS) between male and female metro users. However, compared with female metro users, male counterparts are more likely to hold an attitude that DBS is quicker than walking to connect the metro. Moreover, male metro users also have a higher willingness to carry out some daily physical activities than their female counterparts. Such a result is consistent with the work of Lee [76], which reveals that the female is less likely to be active than the male. Additionally, it is observed that the attitudes toward searching buses and DBS differ between male and female groups for their access trips connecting home and the metro. We found that female metro users think it is easier to take a bus but more difficult to search for DBS bikes around their home than male metro users. It is possible that compared with the female, the male is more likely to overestimate the bus waiting time, which is measured by a ratio of perceived waiting time to actual waiting time [77]. 

### 5.3. MNL Modeling Results

Table 5 and Table 6 present the MNL modeling results for access and egress trips, respectively, using walking as the reference group. The Pseudo R^2^ of *Access* and *Egress* MNL models (0.237 and 0.206) indicate that the MNL models have acceptable goodness of fit. Obviously, the MNL models can explain more variances in the access integrated use than the egress integrated use. 

#### 5.3.1. The Role of Perceived Traffic Safety

The vehicle-related safety concern has significant effects on the feeder mode choice, while bicycle-related safety risk does not play such a significant role. The perception of bicycle-related crashes only affects mode choice between DBS and walking for access trips. A one-unit increase in the score of the perceived bicycle-related crash decreases the odds for choosing DBS relative to walking by 23.6% (= 1–0.764), holding all the other variables constant. 

A higher perceived risk of the vehicle-related crash encourages the choices of DBS and buses rather than walking to/from the metro station. For instance, high-speed vehicles crossing the intersections may make pedestrians feel dangerous. Thus, the exposures to vehicle-related crashes are deterrents for walk–metro users. Bikeway can provide cyclists with safety protection from the vehicle crash, and the bus is a sheltered mode protecting commuters from slight collisions with vehicles. For access trips, we see a 35.4% and 46.4% increase in the odds for opting for DBS and buses, respectively, relative to walking for a one-unit increase in the perceived risk of the vehicle-related crash, while the values for egress trips are 30.2% and 31.6%, respectively. This finding indicates that metro passengers are more affected by vehicle-related safety risks for access trips than for egress trips.

#### 5.3.2. The Role of Attitude

Specific attitudes toward transport modes are observed to have an essential role in determining mode choice. As many previous studies concluded, bike-sharing mostly replaces walking for travel [78,79]. Our results reveal that a positive attitude toward DBS and cycling behavior but a negative attitude toward walking can significantly promote the likelihood of adopting DBS rather than walking as the feeder mode. More specifically, every one-unit higher in the score of cycling attitude can increase the odds for choosing DBS relative to walking by 87.8% (= 1.878–1) for access trips and 27.6% (= 1.276–1) for egress trips; a one-unit increase in the DBS attitude score increased the odds for opting for DBS relative to walking by 127.9% (= 2.279–1) and 60.9% (= 1.609–1) for access and egress trips, respectively. However, the odds for adopting DBS relative to walking will decrease by more than 30% for each unit in an increase in the walking–attitude score (OR = 0.700 in the *Access* model and OR = 0.647 in the *Egress* model). Similarly, by comparing the coefficients of the same variable, we find that favorable attitudes toward DBS and cycling behavior also promote the substitution effect of DBS to buses as the feeder mode. Moreover, it is found that the attitude toward buses does not significantly affect mode choice. We only observe that a negative attitude toward walking increases the possibility of choosing buses for an access trip, particularly when the bus service is easily perceived to be offered around the home. This observation is reasonable.

Relative attitudes between travel modes are also crucial to metro passengers’ feeder mode choice. If metro users think that DBS is faster than walking to connect the metro station, they will have a higher possibility of adopting DBS as the feeder mode (OR = 1.631 in the *Access* model and OR = 1.921 in the *Egress* model). Moreover, a relative attitude that DBS is quicker than buses can increase the odds of choosing DBS as an egress mode. Our results are consistent with the study by Heinen and Bohte [18]. 

Table 5 and Table 6 also show significant effects of the attitude toward DBS/bus availability on the feeder mode choice, mostly applicable for the access trip scenario. It shows that, for access trips, an attitude of easy access to the bus stop can add the willingness to take buses rather than walk for connecting the metro. Moreover, a positive attitude of searching for DBS bike around the home significantly increases the odds of DBS–metro integration. This outcome indicates a self-reinforcing effect in terms of the attitudes toward the DBS/bus availability. Furthermore, the perception of ease of searching for DBS bikes is crucial for replacing walking with DBS for both access and egress trips. However, the attitude toward physical activity is insignificant in affecting the feeder mode choice.

#### 5.3.3. The Role of Socio-Demographic Characteristics

The socio-demographic characteristics are strongly related to the feeder mode choice. This outcome is in line with existing literature [22,23,80]. More specifically, our results show that DBS is preferable for males than females to finish their access trips, while no significant difference exists between genders for egress trips. Age and income significantly affect the feeder mode choice of egress trips, but not that of access trips. Compared to those under 25 years, young adults (26 to 35 years) have a higher willingness to opt for DBS relative to walking (OR = 1.745) in egress trips. However, older adults prefer using buses (relative to walking) for egress trips. Interestingly, we found that middle- and high-incomers (monthly income > 5000 RMB) are more likely to walk than taking buses as their major mode for access trips. Buses are usually crowded during peak hours and lack privacy, which is what high earners care about. 

Table 5 shows that, compared with urban peers, suburban respondents are more likely to transfer by buses than walking for the access trip (home-to-metro); and that compared with suburban residents, urban peers are more likely to use DBS than walking for the access trip. The two findings are reasonable. On the one hand, metro accessibility is lower in suburban areas, and access distance to the metro station is longer. Suburban commuters cannot reach the metro by walking as their urban peers do, so buses are preferable for them (relative to walking). On the other hand, cycling infrastructures are more developed in urban areas than in suburban areas, leading to a higher willingness to transfer by DBS. Thus, DBS is more prevalent in cities than in suburban areas. 

Table 6 indicates that for egress trips (metro-to-workplace), walking is more likely adopted in urban areas, while DBS and buses are more prevalent in suburban areas. Three possible reasons could be proposed. First, in urban areas, the dense distribution of the metro system in urban areas makes it unnecessary to transfer by DBS and buses because of the short transfer distance. Second, the heavy traffic condition in urban areas (e.g., traffic congestion) may be perceived to be unsafe by cyclists, whereas riding buses is time-consuming and unnecessary in most cases in urban areas of Shenzhen, a quintessential transit-dependent city. Third, the high quality of pedestrian infrastructures in urban areas means high walkability, which is friendly to pedestrians.

Moreover, transfer distance is significantly associated with the feeder mode choice, which is in agreement with previous studies [16,23]. Our results also show that DBS and buses are attractive for long-distance trips in the two scenarios. Additionally, the coefficient of the variable *Transfer distance* in the *Bus* model is larger than that in the *DBS* model, indicating that the substitution effect between bus and walking is more significant than that between DBS and walking when the transfer distance is reasonably long. 

## 6. Discussion

Like many other transit modes, the metro offers commuters stop-to-stop services instead of door-to-door services. First- and last-mile issues are, therefore, inevitable for metro trips [10]. A synergy between the metro and other travel modes, either motorized (e.g., bus and car) or non-motorized (e.g., walking and cycling), brings potentials to promote urban mobility through addressing the first- and last-mile problem [11,21]. Identifying the determinants of the feeder mode choice is, therefore, dispensable for understanding the first-/last-mile behavior of metro commuters.

This study contributes to the literature in three aspects. First, we explored the feeder mode choices of metro commuters in the Chinese mega-city context featured with unique characteristics of feeder behaviors, thereby generating diverse targeted implications for improving the seamless connection of metro transit. Second, the discussion of DBS, the newly emerged transport mode that has profoundly reshaped feeder mode choices, enriches the traditional transport research on multimodal behavior. Third, we compared the effects of traffic safety perception and attitudes on the feeder mode choices between access and egress feeder trip scenarios, which have been scarcely discussed in the literature. 

This study provides a useful reference to guide metro users to choose reasonable feeder modes for connecting the metro transit with optimal utilities. Its findings also provide DBS/bus operators and the local government with a valuable reference for the management and improvement of the first- and last-mile. For instance, setting sideways and exclusive bikeways may improve safety perception, thereby encouraging active travel modes (e.g., walking and cycling) for connecting the metro transit [24]. Appropriate distributions of DBS bikes and bus stops near origins/destinations in metro catchment areas and close to metro entrances/exits are indispensable for fostering a good attitude toward DBS/bus usage [16]. During peak hours at metro entrances/exits, DBS bikes are excessively allocated and parked disorderly, whereas the queue for the bus is usually long, clogging the road. Thus, effective management for metro connection around metro entrances/exits is necessarily provided by local transport departments or bureaus. Moreover, available feeder services (e.g., bus lines, fare discount scheme, smart card, and real-time information system) and people-friendly facilities (e.g., bicycle parking space, protected shelters, benches, sidewalks, and exclusive bikeways) in metro catchment areas are suggested to be offered by local transport departments or bureaus [24,38]. More attention and efforts should be paid to suburban areas where feeder services and facilities are less equipped. These measures aiming at a seamless metro transit connection contribute to promoting metro usage or facilitating the modal shift from the car to the metro transit, thereby benefiting sustainable development urban transport. Furthermore, in today’s era with diversified last-mile travel choices, we hope that our topic can ignite a tremendous fascination from local and international researchers.

However, there are some limitations that deserved future research. First, limited by the questionnaire design, we only include two traffic safety variables (bicycle crash and vehicle crash) in the MNL models. As such, more perceived safety factors such as in-vehicle safety in buses and cars should be considered in future studies [43]. Second, attitude factors are insufficiently considered. For example, the attitudes toward economic cost and environmental awareness, which this study fails to capture, may affect the decision-making process of feeder trip mode choice. Third, attitudes may be shaped by objective, physical factors. For example, the attitude towards bus/PBS availability is likely related to the objective level of service. In a similar vein, perceived traffic safety is possibly associated with the actual number of accidents. As such, exploring the interplay between objective factors and (subjective) attitudes (e.g., objectively measured and perceived service quality) is worthy of examination. Fourth, future studies can be devoted to exploring the relationships between traffic safety, attitude, socio-demographic characteristics, and feeder mode choice by revealing the underlying mediation or moderation effects. Last but not least, as travel behavior is jointly shaped by socio-economic variables, the physical environment (built environment + natural environment), and perceptions or attitudes, examining the relative importance of all the independent variables and determining which category plays a larger role in shaping travel behavior is worthy of investigation. Machine learning techniques (e.g., support vector machine, decision tree, random forest, gradient boosting decision tree, and extreme gradient boosting model) are recommended to be adopted in future studies.

## 7. Conclusions

In a departure from existing literature, this study explores how perceived traffic safety and attitude factors are associated with metro commuters’ feeder mode choice during the morning peak time. Our analysis results basically answer our four research questions (see Section 1) and can be listed as follows. (1) Walking is the most frequently used mode for connecting the metro (accounting for over 70%), followed by DBS and buses. The high feeder mode share of walking and DBS is unique in the context of Mainland Chinese mega cities, which differs from European and North American cities; (2) Variances in traffic safety perception and attitude exist across gender and space; (3) The variance in the attitude toward the feeder mode between genders is minimal (or subtle), but men’s attitude toward the DBS/bus availability remarkably differs from women’s; (4) The vehicle-related crash risk usually discourages walking but supports the DBS and buses as transfer modes, whereas the bicycle-related crash is a barrier of transfer by DBS for access trips; (5) Positive attitudes toward cycling and DBS make DBS competitive as a feeder mode. A good attitude toward walking promotes walk–metro integration, but the attitude toward buses does not matter in the feeder mode choice; and (6) Perceived traffic safety and attitudes toward the mode play different roles in shaping first- and last-mile mode choices.

## Figures and Tables

**Table 1:** Measurements and statistics of variables of safety, attitude, and socio-demographic characteristics (*N* (access trips) = 1086; *N* (egress trips) = 1108).

Variable	Description	Category and/or Code	Mean/Percentage	
			Access Trip	Egress Trip
Explanatory variables: safety and attitude				
Bicycle crash	I have safety concerns about crashes with a bicycle along the feeder trip.	Strongly disagree = 1; Somewhat disagree = 2; Somewhat agree = 3; and Strongly agree = 4	3.04	3.15
Vehicle crash	I have safety concerns about crashes with a vehicle along the feeder trip.		2.45	2.43
Cycling	I like to ride a bicycle.		3.09	3.09
Walking	I like to walk.		3.15	3.14
Bus	I like to take a bus.		2.34	2.33
DBS	I like DBS.		2.97	2.97
DBS vs. walking	I think DBS is quicker than walking to connect the metro.		2.80	2.81
DBS vs. bus	I think DBS is quicker than buses to connect the metro.		2.76	2.77
Easy to take a bus	I think it is easy to take a bus to connect the metro.		2.95	2.88
Easy to find DBS	I think it is easy to search for a DBS bike to connect the metro.		2.29	2.60
Physical activity	I would like to have daily physical activities.		3.14	3.14
Control variables: socio-demographic characteristics				
Gender	Male or female	Female	41.16%	41.34%
		Male	58.84%	58.66%
Age	/	<25 years	32.23%	32.13%
		26 to 35 years	51.66%	52.08%
		36 to 45 years	12.80%	12.73%
		>46 years	3.31%	3.07%
Education	Education status	Middle school or below	1.93%	1.99%
		High school	9.85%	10.11%
		University/College	75.32%	75.00%
		Graduate institute	12.89%	12.91%
Income	Monthly personal income	<4999 RMB	11.97%	11.82%
		5000 to 9999 RMB	44.94%	44.68%
		10,000 to 14,999 RMB	23.39%	23.29%
		>15,000 RMB	19.71%	20.22%
Bicycle ownership		No	89.32%	88.36%
		Yes	10.68%	11.64%
Location	Location of the feeder trip	Urban area	43.46%	79.69%
		Suburban area	56.54%	20.31%
Transfer distance	The Euclidean distance of the trip (km)		0.766	0.565

**Table 2:** Feeder mode choice of men and women.

Mode	Access Trip		Egress Trip	
	Female	Male	Female	Male
Walking	72.71%	70.27%	76.64%	75.85%
DBS	11.41%	17.21%	13.54%	15.23%
Bus	15.88%	12.52%	9.83%	8.92%

**Table 3:** Feeder mode choice in urban and suburban areas.

Mode	Access Trip		Egress Trip	
	Urban Area	Suburban Area	Urban Area	Suburban Area
Walking	75.85%	67.75%	80.29%	60.00%
DBS	16.31%	13.68%	12.68%	21.78%
Bus	7.84%	18.57%	7.02%	18.22%

**Table 4:** Two-sample t-test results of individual variance in perceived traffic safety and attitude.

Variable	Male vs. Female Passengers			Urban vs. Suburban Location		
	Difference of Mean	*F*	*Sig.*	Difference of Mean	*F*	*Sig.*
Access feeder trip (home-side, *N* = 1086)						
Bicycle crash	0.049	1.120	0.290	0.210 ***	21.074	0.000
Vehicle crash	−0.071 *	2.003	0.097	0.152 ***	9.311	0.002
Cycling	0.051	1.193	0.275			
Walking	0.038	0.613	0.434			
Bus	−0.004	0.008	0.929			
DBS	0.028	0.457	0.499			
DBS vs. walking	0.162 ***	10.068	0.002			
DBS vs. bus	0.030	0.339	0.561			
Easy to take a bus	−0.111 **	4.371	0.037			
Easy to find DBS	0.113 **	4.299	0.038			
Physical activity	0.119 ***	11.400	0.001			
Egress feeder trip (workplace-side, *N* = 1108)						
Bicycle crash	0.040	0.813	0.367	−0.124 **	5.169	0.023
Vehicle crash	−0.103 **	4.031	0.045	−0.187 ***	8.960	0.003
Cycling	0.044	0.875	0.350			
Walking	0.038	0.630	0.427			
Bus	−0.003	0.004	0.947			
DBS	0.034	0.665	0.415			
DBS vs. walking	0.146 ***	8.147	0.004			
DBS vs. bus	0.045	0.754	0.385			
Easy to take a bus	0.007	0.017	0.897			
Easy to find DBS	0.049	0.785	0.376			
Physical activity	0.123 ***	12.462	0.000			

Note: *** *p* < 0.01; ** *p* < 0.05; * *p* < 0.1.

**Table 5:** Results of the access MNL model (reference group: walking, *N* = 1086).

Variable	DBS				Bus			
	Coef.	Odds Ratio	Std.	z	Coef.	Odds Ratio	Std.	z
Safety and attitude variables								
Bicycle crash	−0.269 **	0.764	0.146	−1.99	0.028	1.028	0.135	0.19
Vehicle crash	0.303 **	1.354	0.135	2.39	0.381 ***	1.464	0.127	2.82
Cycling	0.630 ***	1.878	0.164	3.84	0.069	1.071	0.164	0.42
Walking	−0.357 ***	0.700	0.148	−2.60	−0.285 *	0.752	0.138	−1.92
Bus	−0.018	0.982	0.282	−0.07	0.318	1.374	0.258	1.13
DBS	0.824 ***	2.279	0.180	4.14	−0.213	0.809	0.199	−1.18
DBS vs. walking	0.489 ***	1.631	0.158	3.13	0.277 *	1.319	0.156	1.75
DBS vs. bus	−0.028	0.972	0.183	−0.16	−0.260	0.771	0.171	−1.42
Easy to take a bus	−0.396 **	0.673	0.201	−2.36	0.599 ***	1.821	0.168	2.98
Easy to find DBS	0.605 ***	1.832	0.129	5.05	−0.098	0.906	0.120	−0.76
Physical activity	−0.263	0.768	0.192	−1.38	−0.153	0.858	0.191	−0.80
Control variables								
Gender (reference: female)								
Male	0.342 *	1.407	0.222	1.59	−0.181	0.835	0.215	−0.81
Age (reference: under 25 years)								
26–35 years	0.187	1.206	0.263	0.78	0.138	1.148	0.241	0.53
36–45 years	−0.468	0.626	0.358	−1.28	0.366	1.442	0.367	1.02
Over 46 years	−0.387	0.679	0.644	−0.63	−0.015	0.985	0.613	−0.02
Education (reference: middle school or below)								
High school	−0.137	0.872	0.758	−0.19	−0.379	0.684	0.728	−0.50
College/University	−0.276	0.759	0.718	−0.40	−0.573	0.564	0.694	−0.80
Graduate institute	−0.523	0.593	0.786	−0.69	−0.419	0.658	0.753	−0.53
Income (reference: <4999 RMB)								
5000 to 9999 RMB	0.132	1.141	0.323	0.39	−0.608 *	0.544	0.336	−1.89
10,000 to 14,999 RMB	0.206	1.229	0.401	0.54	−1.134 ***	0.322	0.379	−2.83
>15,000 RMB	−0.239	0.788	0.413	−0.56	−0.781 *	0.458	0.423	−1.89
Bicycle ownership (reference: no)								
Yes	0.203	1.225	0.322	0.65	0.600 *	1.822	0.314	1.86
Home location (reference: urban area)								
Suburban area	−0.351 *	0.704	0.236	−1.69	0.397 *	1.487	0.208	1.68
Transfer distance	0.972 ***	1.001	0.158	5.98	1.298 ***	1.001	0.163	8.21
Intercept	−6.378 ***	0.002	1.249	−5.16	−3.678 ***	0.025	1.237	−2.95
Pseudo R^2^	0.237							
Log-likelihood	−661.507							

Note: *** *p* < 0.01; ** *p* < 0.05; * *p* < 0.1.

**Table 6:** Results of the egress MNL model (reference group: walking, *N* = 1108).

Variable	DBS				Bus			
	Coef.	Odds Ratio	Std.	z	Coef.	Odds Ratio	Std.	z
Safety and attitude variables								
Bicycle crash	−0.186	0.830	0.163	−1.38	−0.089	0.915	0.135	−0.55
Vehicle crash	0.264 **	1.302	0.144	2.13	0.275 *	1.316	0.124	1.91
Cycling	0.244 *	1.276	0.181	1.65	−0.015	0.985	0.147	−0.08
Walking	−0.435 ***	0.647	0.163	−3.23	−0.255	0.775	0.135	−1.56
Bus	0.029	1.029	0.322	0.11	0.140	1.150	0.259	0.43
DBS	0.475 ***	1.609	0.200	2.60	0.068	1.070	0.183	0.34
DBS vs. walking	0.653 ***	1.921	0.170	4.02	0.079	1.082	0.162	0.47
DBS vs. bus	0.428 **	1.535	0.201	2.42	−0.127	0.881	0.177	−0.63
Easy to take a bus	−0.202	0.817	0.204	−1.27	0.066	1.069	0.160	0.33
Easy to find DBS	0.261 **	1.298	0.135	2.35	0.053	1.055	0.111	0.40
Physical activity	0.236	1.266	0.213	1.26	0.047	1.048	0.187	0.22
Control variables								
Gender (reference: female)								
Male	0.141	1.152	0.246	0.68	−0.034	0.967	0.208	−0.14
Age (reference: under 25 years)								
26–35 years	0.557 **	1.745	0.296	2.32	−0.015	0.986	0.240	−0.05
36–45 years	0.387	1.473	0.385	1.14	0.791 **	2.207	0.340	2.06
Over 46 years	0.302	1.353	0.570	0.51	1.026 *	2.789	0.594	1.80
Education (reference: middle school or below)								
High school	−0.321	0.725	0.697	−0.48	−1.021	0.360	0.671	−1.46
College/University	−0.996	0.369	0.647	−1.56	−1.381 **	0.251	0.638	−2.13
Graduate institute	−1.419 *	0.242	0.754	−1.94	−1.299 *	0.273	0.730	−1.72
Income (reference: <4999 RMB)								
5000 to 9999 RMB	0.028	1.029	0.367	0.09	−0.219	0.803	0.313	−0.60
10,000 to 14,999 RMB	−0.502	0.605	0.444	−1.36	−0.589	0.555	0.368	−1.33
>15,000 RMB	−1.180 ***	0.307	0.476	−2.78	−0.706	0.493	0.425	−1.48
Bicycle ownership (reference: no)								
Yes	0.215	1.240	0.341	0.72	0.406	1.500	0.298	1.19
Workplace location (reference: urban area)								
Suburban area	0.445 *	1.560	0.272	1.91	0.489 *	1.630	0.233	1.80
Transfer distance	1.143 ***	1.001	0.194	5.96	1.605 ***	1.002	0.192	8.29
Intercept	−6.796 ***	0.001	1.275	−5.42	−2.282 *	0.102	1.255	−1.79
Pseudo R^2^	0.206							
Log-likelihood	−622.990							

Note: *** *p* < 0.01; ** *p* < 0.05; * *p* < 0.1.
